# Combination of an anti-EGFRvIII antibody CH12 with Rapamycin synergistically inhibits the growth of EGFRvIII^+^PTEN^−^ glioblastoma *in vivo*

**DOI:** 10.18632/oncotarget.8407

**Published:** 2016-03-26

**Authors:** Wen Xu, Yanyu Bi, Juan Kong, Jiqin Zhang, Biao Wang, Kesang Li, Mi Tian, Xiaorong Pan, Bizhi Shi, Jianren Gu, Hua Jiang, Xianming Kong, Zonghai Li

**Affiliations:** ^1^ Medical School of Fudan University, Shanghai, China; ^2^ State Key Laboratory of Oncogenes and Related Genes, Shanghai Cancer Institute, Renji Hospital, Shanghai Jiaotong University School of Medicine, Shanghai, China; ^3^ Renji Hospital, Medical School of Shanghai Jiaotong University, Shanghai, China

**Keywords:** EGFRvIII^+^PTEN^−^ GBM, CH12, rapamycin, STAT5

## Abstract

There are still unmet medical needs for the treatment of glioblastoma (GBM), the most frequent and aggressive brain tumor worldwide. EGFRvIII, overexpressed in approximately 30% of GBM, has been regarded as a potential therapeutic target. In this study, we demonstrated that CH12, an anti-EGFRvIII monoclonal antibody, could significantly suppress the growth of EGFRvIII^+^ GBM *in vivo*; however, PTEN deficiency in GBM reduced the efficacy of CH12 by attenuating its effect on PI3K/AKT/mTOR pathway. To overcome this problem, CH12 was combined with the mTOR inhibitor rapamycin, leading to a synergistic inhibitory effect on EGFRvIII^+^PTEN^−^ GBM *in vivo*. Mechanistically, the synergistic antitumor effect was achieved via attenuating EGFR and PI3K/AKT/mTOR pathway more effectively and reversing the STAT5 activation caused by rapamycin treatment. Moreover, the combination therapy suppressed angiogenesis and induced cancer cell apoptosis more efficiently. Together, these results indicated that CH12 and rapamycin could synergistically suppress the growth of EGFRvIII^+^PTEN^−^ GBM, which might have a potential clinical application in the future.

## INTRODUCTION

GBM is the most common and malignant subtype of primary brain tumors, accounting for 54.4% of primary brain and CNS gliomas [1] and exhibiting extremely aggressive biological behavior, with the progressive invasion of wide areas of the brain parenchyma [2]. The current standard of care combines maximal surgical resection, followed by radiotherapy with concomitant and adjuvant temozolomide [3]. Despite this multimodal approach, median survival is limited to 16 to 19 months, with approximately 25% to 30% of the patients alive 2 years after diagnosis [3–5].

GBM consists of a genetically and phenotypically heterogeneous group of tumors [6]. Ninety percent of GBM cases develop *de novo* (primary glioblastoma) from normal glial cells via multistep tumorigenesis. The remaining 10% of GBM cases are secondary neoplasms, developing through progression from low-grade tumors (diffuse or anaplastic astrocytomas), which takes approximately 4–5 years. Secondary GBM grows more slowly and has a better prognosis than the *de novo* case, which develops within 3 months [7, 8]. Although these two types show no morphological differences, the genetic basis and the molecular pathways are different [6], with TP53 mutations occurring more commonly in secondary GBM and EGFR amplifications and PTEN mutations occurring more frequently in primary GBM [6, 9].

Overall, the aberrant amplification, deletion or mutation of at least one receptor tyrosine kinase (RTK) has been found in 67.3% of GBM, with EGFR accounting for 57.4% [10]. Importantly, approximately 50% of patients with EGFR amplification harbor a specific mutation known as EGFR variant III (EGFRvIII, de2-7EGFR), which is characterized by the deletion of exon 2–7, resulting in an in-frame deletion of 267 amino acid residues from the extracellular domain [11, 12]. This deletion generates a receptor that is unable to bind a ligand, yet is constitutively, but weakly, active [13]. Continuous, low-level activation leads to impaired internalization and degradation of the receptor, causing prolonged signaling [14]. EGFRvIII has been identified in GBM, lung, ovarian, breast cancers, and glioma, but has never been identified in normal tissue [15, 16], correlating with poor prognosis in the clinic [17, 18]; therefore, it is an attractive therapeutic target. Monoclonal antibodies (mAbs), including mAb806 and CH12 (a mAb developed in our lab), which could selectively bind to EGFRvIII have been demonstrated to be capable of efficiently suppressing the growth of EGFRvIII-positive tumor xenografts [19, 20]. Additionally, in a phase I study, ch806 (a chimeric antibody derived from mAb806) displayed significant accumulation in cancer tissues without definite uptake in normal tissues [21].

PTEN is a lipid phosphatase with a canonical role in turning-off PI3K/AKT/mTOR signaling [22], a pathway of the RTK downstream signal (including the EGFR family), which plays important roles in regulating tumor proliferation, differentiation, migration and survival [23, 24]. PTEN is deleted in 50%–70% of primary GBM and 54%–63% of secondary cases, and it is also mutated in 14%–47% of primary cases [25]. Co-expression of EGFRvIII and PTEN was significantly associated with a clinical response to EGFR inhibitors [26]. PTEN deficiency causes the activation of PI3K/AKT/mTOR pathway and leads to the resistance to EGFR inhibitors and the overall survival of patients shortening [23, 24]. Therefore, the inhibition of the mTOR signaling pathway has been considered to be an attractive treatment strategy for PTEN^−^ GBM [24, 27].

Rapamycin and its analogs have demonstrated efficacy in GBM by inhibiting the mTOR pathway and inactivating the vital downstream kinases, the p70S6 kinase and the eukaryotic initiation factor 4E binding protein-1(4E-BP-1) [28]; however, most clinical trials using inhibitors of the components in this pathway as monotherapies have failed to demonstrate survival benefit in glioblastoma patients [29]. For instance, temsirolimus, a dihydroxymethyl propionic acid ester of rapamycin, suggested initial disease stabilization in approximately 50% of patients, but the durability of response was short because of the narrow safety window [30]. It is worth determining whether combining the anti-EGFRvIII antibody CH12 with rapamycin might reduce the dose of rapamycin necessary or boost its efficacy in EGFRvIII^+^PTEN^−^ GBM. Therefore, in this study, we evaluated the efficacy of rapamycin and CH12 monotherapy and the combination in EGFRvIII^+^PTEN^−^ GBM and elucidated the molecular mechanisms underlying their antitumor effects.

## RESULTS

### CH12 significantly suppressed the growth of EGFRvIII^+^PTEN^−^ glioblastoma *in vivo*

**Figure 1:**
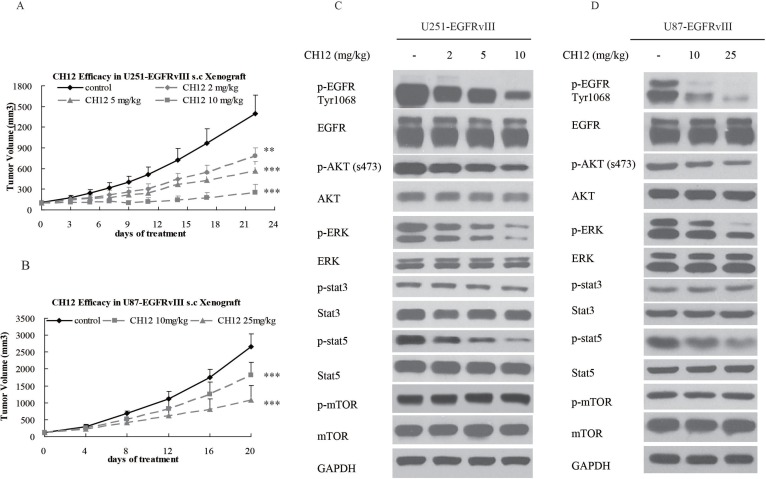
CH12 significantly suppressed the growth of EGFRvIII^+^PTEN^−^ glioblastoma *in vivo* (**A**) Growth curve of U251-EGFRvIII xenografts treated intraperitoneally by control (vehicle) or CH12 at concentrations of 2, 5, or 10 mg/kg three times per week for 2 weeks. (**B**) Growth curve of U87-EGFRvIII xenografts treated intraperitoneally by control (vehicle), or CH12 at concentrations of 10 or 25 mg/kg three times per week for 2 weeks. (**C, D**) The changes in relevant signaling pathways in U251-EGFRvIII and U87-EGFRvIII xenografts were assessed by Western blot after CH12 treatment. The data are expressed as the mean tumor volumes ± SE. Statistical significance is indicated versus control (*P < 0.05, **P < 0.01, ***P < 0.001).

To determine the efficacy of CH12 in EGFRvIII^+^PTEN^−^ glioblastoma, the nude mice bearing U251-EGFRvIII and U87-EGFRvIII s.c. xenografts were treated with CH12 i.p. three times a week for 2 weeks. As shown in Figure 1A and 1B, CH12 significantly inhibited the growth of U251-EGFRvIII and U87-EGFRvIII xenografts in a dose-dependent manner. We investigated the mechanisms underlying the reduction in tumor proliferation caused by CH12 treatment. The results (Figure 1C and 1D) showed that CH12 attenuated the phosphorylation of EGFR, AKT and ERK, and that it also inhibited the phosphorylation of STAT5, a transcription factor, regulating gene expression when stimulated by a wide variety of growth factors, hormones, and cytokines and contributing to proliferation in human GBM tumors [31–32]. The phosphorylation of AKT, however, was not totally inhibited, and no inhibitory effect was found in mTOR pathway. Together, CH12 markedly diminished the growth of EGFRvIII^+^PTEN^−^ glioblastoma *in vivo* via inhibiting EGFR and STAT5 pathway but had no effect in mTOR pathway.

### Rapamycin inhibited the growth of EGFRvIII^+^PTEN^−^ glioblastoma *in vivo*

**Figure 2:**
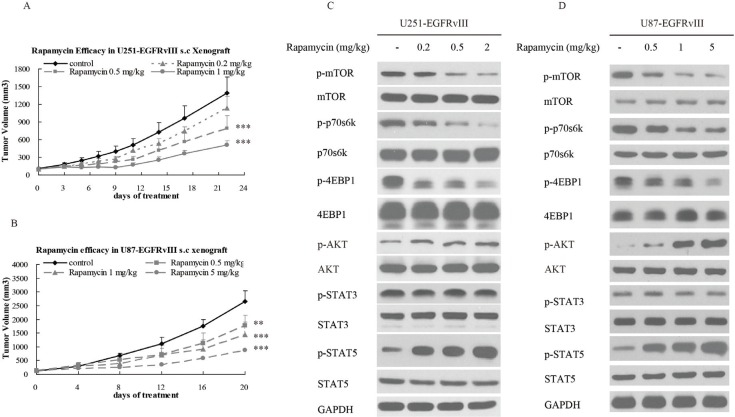
Rapamycin inhibited the growth of EGFRvIII^+^PTEN- glioblastoma *in vivo* (**A**) Growth curve of U251-EGFRvIII xenografts treated intraperitoneally with control (vehicle) or rapamycin at concentrations of 0.2, 0.5, or 1 mg/kg four times per week for 2 weeks. (**B**) Growth curve of U87-EGFRvIII xenografts treated intraperitoneally with control (vehicle) or rapamycin at concentrations of 0.5, 1, or 5 mg/kg four times per week for 2 weeks. (**C, D**) The changes in relevant signaling pathways in U251-EGFRvIII and U87-EGFRvIII cells were assessed by Western blot after rapamycin treatment. The data are expressed as the mean tumor volumes ± SE. Statistical significance is indicated versus control (*P < 0.05, **P < 0.01, ***P < 0.001).

To determine the antitumor efficacy of rapamycin in EGFRvIII^+^PTEN^−^ GBM, nude mice bearing U87-EGFRvIII and U251-EGFRvIII xenografts were treated with rapamycin i.p. four times a week. The results demonstrated that rapamycin significantly inhibited tumor growth (Figure 2A and 2B). The mechanism study suggested that rapamycin inhibited the phosphorylation of mTOR, p70s6k and 4E-BP-1, but it also activated STAT5 and AKT (Figure 2C and 2D). This implies that the individual inhibition of EGFR, STAT5 and PI3K/AKT/mTOR pathway at once a time be a potential therapeutic strategy.

### Combination of CH12 with rapamycin synergistically inhibited the growth of the EGFRvIII^+^PTEN^−^ glioblastoma xenografts

**Figure 3:**
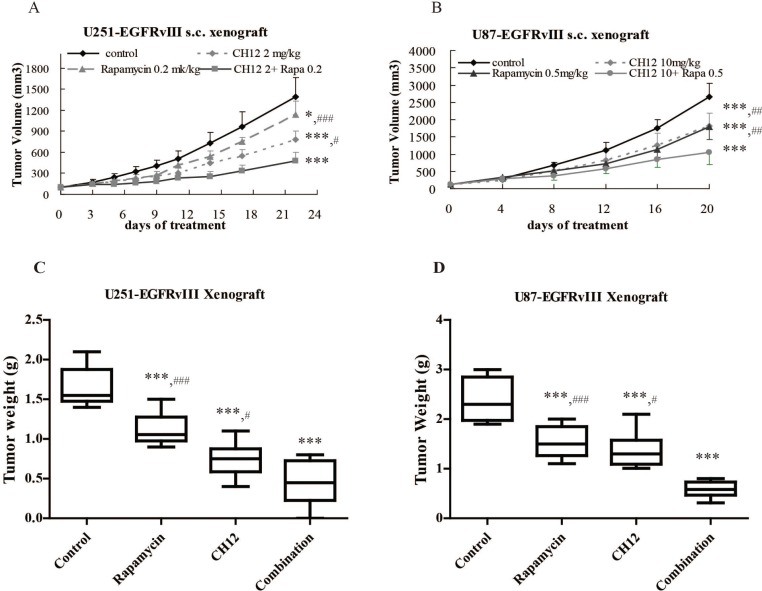
Combination of CH12 with rapamycin synergistically inhibited the growth of EGFRvIII^+^PTEN^−^ glioblastoma xenografts Mice bearing U251-EGFRvIII xenografts were treated i.p. with control (vehicle), rapamycin at a concentration of 0.2 mg/kg four times per week, CH12 at a concentration of 2 mg/kg three times per week or the combination of rapamycin and CH12 at the doses used in monotherapy for 2 weeks. Mice bearing U87-EGFRvIII xenografts were intraperitoneally treated with control (vehicle), rapamycin at a concentration of 0.5 mg/kg four times per week, CH12 at a concentration of 10 mg/kg three times per week or the combination for 2 weeks. (**A**) Growth curve of U251-EGFRvIII xenografts. **(B)** Growth curve of U87-EGFRvIII xenografts. (**C**) Tumor weight of U251-EGFRvIII xenografts at the end of the experiment. (**D**) Tumor weight of U87-EGFRvIII xenografts at the end of the experiment. All data are presented as the mean ± SE. *P* < 0.05 was considered statistically significant. *P < 0.05, **P < 0.01, ***P < 0.001 versus the control, ^#^*P* < 0.05, ^##^*P* < 0.01, ^###^*P* < 0.001 versus the combination group.

To investigate the *in vivo* antitumor effect of the combination of CH12 with rapamycin, mice bearing U251-EGFRvIII and U87-EGFRvIII s.c. xenografts were treated with rapamycin, CH12 or the combination. All animals tolerated the treatments without observable signs of toxicity and had stable body weights during the study. The inhibitory ratios of rapamycin at 0.2 mg/kg, CH12 at 2 mg/kg and the combination of rapamycin and CH12 on day 21 after the first administration were 19.6%, 44.0%, and 65.7% in U251-EGFRvIII xenograft model, respectively (Figure 3A); And that of rapamycin at 0.5 mg/kg, CH12 at 10 mg/kg and the combination on day 21 were 32.8%, 31.5%, and 60.3% in U87-EGFRvIII xenograft model, respectively (Figure 3B), which suggested that tumor growth was synergistically inhibited by the combination treatment (*P* < 0.01 versus rapamycin or CH12 treatment alone, CDI < 1). Tumor weight was measured at the end of the study (Figure 3C and 3D), which also indicated that the combination of CH12 and rapamycin had a synergistic antitumor effect in both GBM xenograft model.

### Combination of CH12 with rapamycin significantly extends the survival of mice bearing EGFRvIII^+^PTEN^−^ intracranial glioblastoma tumors

**Figure 4:**
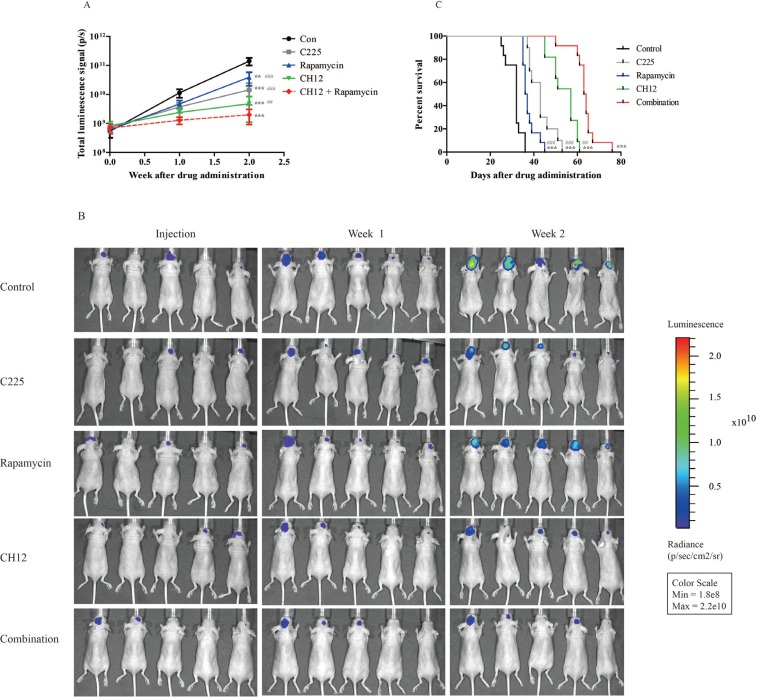
Combination of CH12 with rapamycin synergistically extends the survival of mice bearing EGFRvIII^+^PTEN- intracranial glioblastoma Mice bearing U251-EGFRvIII-luciferase intracranial glioblastoma were treated intraperitoneally with control vehicle, rapamycin at a concentration of 5 mg/kg four times per week, CH12 at a concentration of 25 mg/kg three times per week or the combination for 2 weeks. (**A, B**) Luminescence signals of mice bearing U251-EGFRvIII intracranial glioblastoma after treatment. (**C**) Survival curve of mice bearing U251-EGFRvIII-luciferase intracranial glioblastoma after treatment. The data are expressed as the mean ± SD. *P* < 0.05 was considered statistically significant. *P < 0.05, **P < 0.01, ***P < 0.001 versus the control, ^#^*P* < 0.05, ^##^*P* < 0.01, ^###^*P* < 0.001 versus the combination group.

To assess the potential efficacy of the combination in an intracranial glioblastoma model, we developed U251-EGFRvIII intracranial xenografts labeled with luciferase to image the tumor volume. For 7 days post implantation, the mice were treated with vehicle (control), C225 (cetuximab, a well-known anti-EGFR antibody), CH12, rapamycin, and the combination of CH12 and rapamycin. The luminescence signal showed that C225, CH12, rapamycin and the combination group inhibited tumor growth, and the combination showed significantly better efficacy than that of monotherapy (Figure 4A and 4B). The control group had a median survival of 32 days, whereas mice treated with C225, CH12, rapamycin, or the combination of CH12 and rapamycin had a median survivals up to 43, 57, 36, and 63.5 days, respectively. Compared with monotherapy, the combination extended the median survival significantly (*P* < 0.001, CH12 or rapamycin versus the combination, Figure 4C). Together, the data indicated that the combination of CH12 with rapamycin could synergistically inhibit the growth of EGFRvIII^+^PTEN^−^ glioblastoma xenografts and markedly extend the survival of these mice.

### Combination of CH12 with rapamycin suppressed EGFR and mTOR pathway and reversed the activation of STAT5 in EGFRvIII^+^PTEN^−^ GBM xenografts

**Figure 5:**
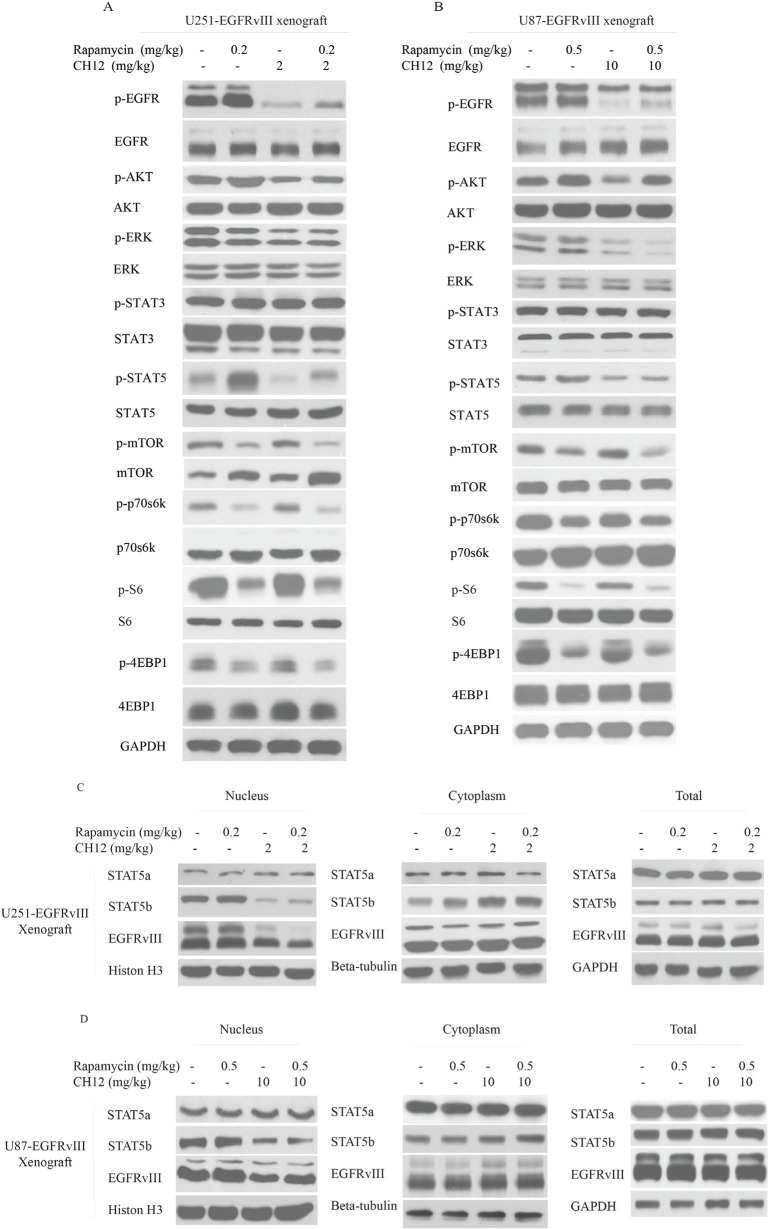
Combination of CH12 with rapamycin suppressed the EGFR and mTOR pathways and STAT5 activation in EGFRvIII^+^PTEN- glioblastoma xenografts (**A**, **B**) The signaling events were evaluated by Western blot upon intraperitoneal treatment with control (vehicle), rapamycin at a concentration of 0.2 mg/kg four times per week, CH12 at a concentration of 2 mg/kg three times per week or the combination for 2 weeks in U251-EGFRvIII cells (A) and treatment with control (vehicle), rapamycin at a concentration of 0.5 mg/kg four times per week, CH12 at a concentration of 10 mg/kg three times per week or the combination for 2 weeks in U87-EGFRvIII cells (B). (**C**, **D**) STAT5b and EGFRvIII nucleus translocation was tested by Western blot following intraperitoneal treatment with control (vehicle), rapamycin, CH12 or the combination for 2 weeks in U251-EGFRvIII (C) and U87-EGFRvIII cells (D).

To gain further insight into the molecular events occurring in the combination-treated tumor xenograft, certain key signaling molecules of EGFR or mTOR pathway were examined by Western blot analysis. The phosphorylation levels of p70s6k and 4E-BP-1 in the combination treatment cells were lower than those in the CH12 groups, and the phosphorylation of STAT5 was also significantly inhibited compared with the rapamycin group (Figure 5A and 5B).

Activated STAT5 dimers bind to specific DNA response elements in the promoter region of target genes in the nucleus and regulate various cellular responses, including growth, migration, survival, and cell motility [31]. To determine the role of STAT5 in rapamycin resistance in EGFRvIII^+^PTEN^−^ GEM, the STAT5 siRNA was applied together with rapamycin to treat GBM. The results illustrated that the combination of STAT5 siRNA and rapamycin significantly enhanced the antitumor effect of rapamycin on U251-EGFRvIII and U87-EGFRvIII cells (Figure S2A and S2B), and STAT5 phosphorylation was significantly inhibited in STAT5 siRNA and the combination group (Figure 2C and 2D). These results further demonstrated that STAT5 activation contributed to rapamycin resistance in EGFRvIII^+^PTEN^−^ GEM.

**Figure 6:**
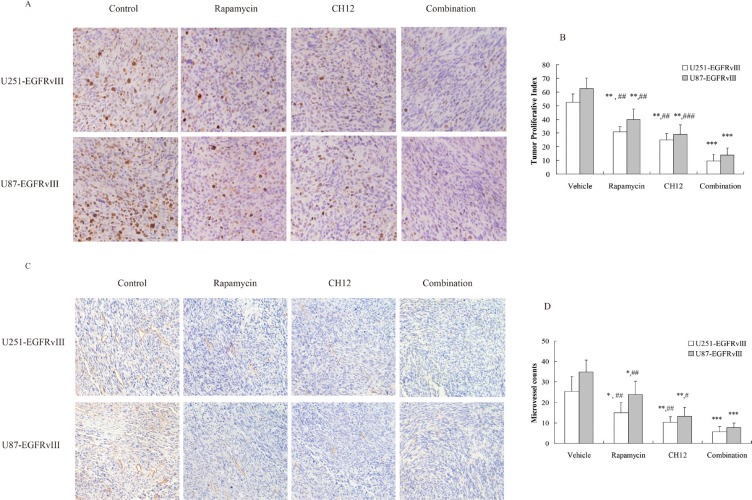

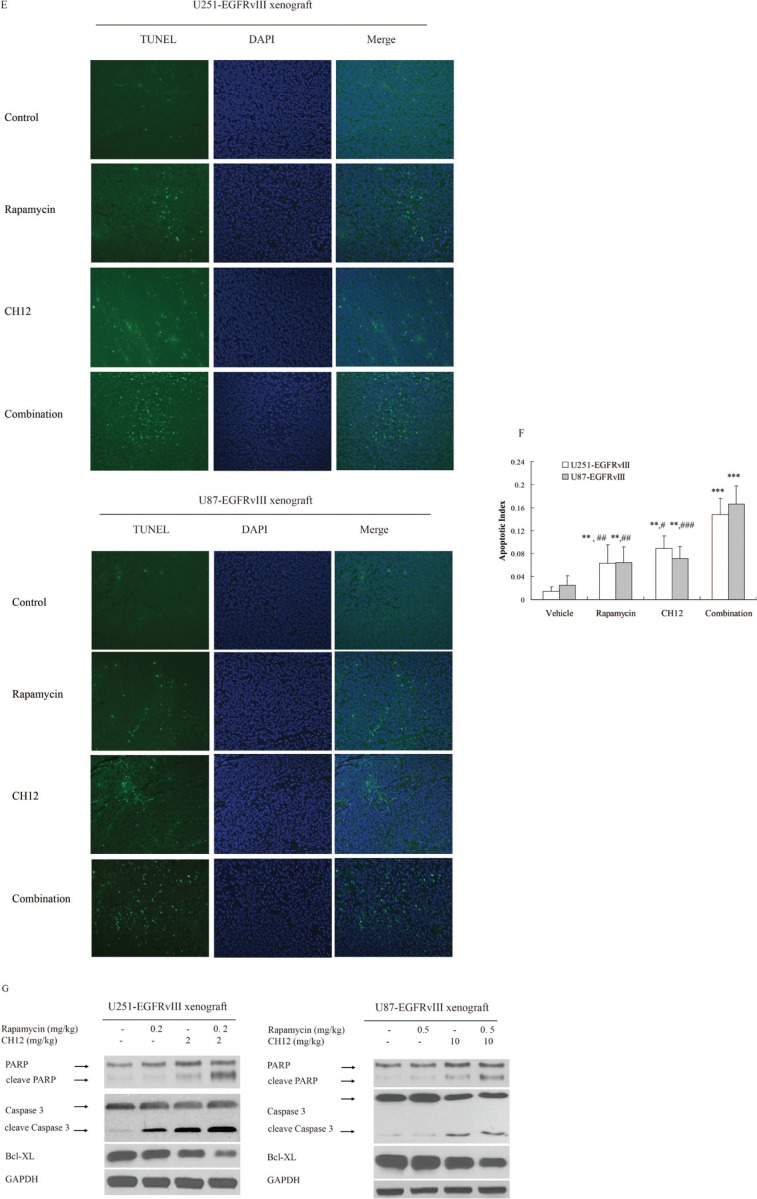
Combination of CH12 with rapamycin reduced proliferation and angiogenesis and induced tumor cell apoptosis (**A**) Combination of CH12 with rapamycin treatment led to less growth compared with controls in U251-EGFRvIII and U87-EGFRvIII xenografts. Tumor sections were stained for Ki-67. The cell proliferative index was assessed as the percentage of total cells that were Ki-67-positive from six randomly selected high-power fields (200 ×) in the xenografts from six mice of each group. (**B**) The qualitative analysis of Ki-67 staining. (**C**) Combination of CH12 with rapamycin led to less vascularization compared with control in U251-EGFRvIII and U87-EGFRvIII xenografts. Tumor sections were immunostained with anti-CD34 antibody. MVD values were analyzed by measuring the number of stained microvessels from six randomly selected fields (200 ×) in the xenografts from six mice of each group. (**D**) The qualitative analysis of CD34 staining. (**E**) Combination of CH12 with rapamycin led to an increase in apoptosis compared with controls in U251-EGFRvIII and U87-EGFRvIII xenografts. Apoptotic cells were detected using the TUNEL assay. The apoptotic index was assessed by the ratio of TUNEL-positive cells : total number of cells from six randomly selected high-power fields (200 ×) in the xenografts from six mice of each group. (**F**) The qualitative analysis of TUNEL assay. (**G**) The signals related to apoptosis were evaluated by Western blot following treatment with CH12, rapamycin or the combination in U251-EGFRvIII and U87-EGFRvIII xenografts. The data are expressed as the mean ± SD. *P* < 0.05 was considered statistically significant. *P < 0.05, **P < 0.01, ***P < 0.001 versus the control, ^#^*P* < 0.05, ^##^*P* < 0.01, ^###^*P* < 0.001 versus the combination group.

It has been reported that the STAT5b/EGFRvIII complex can translocate to the nucleus and promote the translation of the anti-apoptosis protein BCL-XL [33]. Therefore, we examined the nuclear translocation of EGFRvIII and STAT5b. The results showed that combination treatment inhibited their nuclear translocation (Figure 5C and 5D) and reduced the expression of Bcl-XL (Figure 6G). Together, the combination of rapamycin and CH12 inhibited the AKT, ERK and STAT5 pathways more efficiently compared with rapamycin, and the combination treatment inhibited STAT5b and EGFRvIII nuclear translocation and reduced Bcl-XL expression.

### Combination of CH12 with rapamycin potently reduced proliferation and angiogenesis and induced apoptosis in EGFRvIII^+^PTEN^−^ glioblastoma

To further elucidate the causes underlying the *in vivo* synergistic activity of rapamycin and CH12, the proliferative index, tumor microvessel density, and the apoptotic index were evaluated. The proliferative index was significantly lower in the combination-treatment group than the control group and the monotherapy groups (*P* < 0.01 monotherapy versus combination; Figure 6A, 6B). The number of CD34-positive microvessels in the combination-treatment group was also significantly less than in the monotherapy groups (*P* < 0.05 monotherapy versus combination; Figure 6C, 6D). TUNEL staining demonstrated a significant increase in the number of apoptotic cells in the combination-treatment group compared with the monotherapy groups (*P* < 0.01, Figure 6E and 6F). Consistently, cleaved caspase 3 and cleaved PARP increased distinctly, and the expression of Bcl-XL, which typically inhibits apoptosis, was reduced (Figure 6G). Together, these data suggested that rapamycin and CH12 combination therapy had a stronger effect on reducing tumor proliferation and angiogenesis and inducing tumor cell apoptosis, leading to synergistic tumor growth inhibition.

## DISCUSSION

GBM is the most frequent and aggressive form of primary brain tumor. The current standard of care for GBM consists of surgical removal, radiotherapy, and adjuvant chemotherapy (typically temozolomide); however, despite these interventions, the prognosis is still poor, with a mean survival time of 16–19 months following diagnosis [3–5]. EGFRvIII, expressed in approximately 30% of GBM tumors, correlates with poor prognosis in the clinic. The therapeutic antibody directly targeting EGFRvIII suggested significant efficacy in EGFRvIII^+^ GBM xenografts [20]. In recent years, however, drugs targeting EGFR or VEGFR, such as cetuximab, gefitinib, or bevacizumab, have shown limited activity in most clinical trials in GBM patients [34–36]. Thus, it is urgent to find novel treatment strategies to increase the efficacy of the EGFR or VEGFR inhibitor in patients with GBM.

The deficiency of the tumor suppressor gene PTEN in nearly half of GBM cases is a critical cause contributing to EGFR or VEGFR inhibitor-resistance and correlating with the reduced survival of patients, as this is associated with increased activity of the PI3K/Akt/mTOR pathway [23, 26]. Therefore, inhibition of the mTOR signaling pathway has been considered to be an attractive treatment strategy for PTEN^−^ GBM [24, 27].

Considering that EGFRvIII expression in GBM often is accompanied with PTEN deficiency, which abates the efficacy of EGFRvIII inhibitors [37], combining an EGFRvIII inhibitor and an mTOR inhibitor might be a potential strategy for EGFR^+^PTEN^−^ GBM. In this study, CH12 and rapamycin were used alone or combined together to treat EGFRvIII^+^PTEN^−^ GBM. Our results showed CH12 inhibited the tumor growth of U251-EGFRvIII and U87-EGFRvIII xenografts *in vivo* and inhibited EGFR downstream signals, including the phosphorylation of AKT, ERK (partially) and STAT5, but had no effect on the critical mTOR pathway (Figure 1). Rapamycin treatment also delayed the growth of U251-EGFRvIII and U87-EGFRvIII xenografts *in vivo*; however, although the mTOR pathway was inhibited, the phosphorylation of AKT and STAT5 were obviously upregulated (Figure 2). Previous reports suggested that rapamycin lacked effective activity against mTORC2 and induced feedback activation of AKT survival pathways in many tumor types, including GBM [38–40], and the activation of STAT5 by mTOR inhibition was also observed in metastatic breast cancer [41].

Several characteristics associated with GBM malignancies, including invasion, treatment resistance and immunosuppression, have recently been associated with signaling pathways converging on a small number of transcription factors, including the STAT family. STAT5, a latent cytoplasmic protein, has been reported to be associated with the malignant transformation of hematological malignancies, breast cancer, prostate cancer and glioblastoma [32, 42–44]. STAT5 comprises two highly homologous isoforms, STAT5a and STAT5b, which exert not only overlapping, but also distinct functions [45]. STAT5b was identified as the predominant isoform in glioblastoma, particularly in EGFRvIII expressing cells associated with tumor aggressiveness and increased cell invasion [46–48]. STAT5b phosphorylation at Y699 was associated with poor outcome in glioblastoma and interacted with EGFRvIII in the nucleus of glioma cells, and this complex has been found to associate with promoter sequences and regulate gene expression [33]. Importantly, STAT5b is a key regulator of Bcl-XL, which contributes a lot to EGFRvIII-mediated resistance to DNA-damaging chemotherapeutic agents [49]. Accordingly, the knockdown of STAT5b suppressed transformation by EGFRvIII and sensitized glioblastoma cells to cisplatin-induced apoptotic death [49].

Considering the feedback activation of STAT5 by rapamycin, it is reasonable to use CH12, which could suppress the phosphorylation of STAT5, together with rapamycin. The combination of CH12 with rapamycin demonstrated significantly stronger antitumor effects than monotherapy (*P* < 0.01) in EGFRvIII^+^PTEN^−^ s.c. glioblastoma xenografts as well as in an intracranial model. Our intracranial model study also showed that CH12 monotherapy is better than cetuximab. There was a similar report that the anti-EGFRvIII antibody mAb806 had a better antitumor effect than cetuximab in EGFRvIII-positive tumors [50]. The mechanism study suggested combination treatment significantly inhibited the EGFR, PI3k/AKT/mTOR and STAT5 pathways.

Recent reports suggested the use of bevacizumab and everolimus as part of the first-line combined modality therapy for the GBM-improved, progression-free survival (PFS) of patients, compared to previous reports suggesting standard radiation therapy/temozolomide therapy [51]. Moreover, when temsirolimus was used in combination with erlotinib for patients with recurrent malignant gliomas in a phase I/II study, the maximum tolerated dosage of temsirolimus in combination with erlotinib proved to be lower than expected because of increased toxicity [52]. Thus, antitumor activity was not significant, partly, because of insufficient tumor drug levels and redundant signaling pathways. CH12, targeting EGFRvIII, not detected in normal tissue, which increase the safety window markedly; therefore, the synergistic effect was expected.

Taken together, CH12 was combined with rapamycin, leading to a synergistic tumor-suppression effect on EGFRvIII^+^PTEN^−^ glioblastoma *in vivo* via attenuating EGFR and the PI3K/AKT/mTOR pathway more effectively and reversing STAT5 activation caused by rapamycin treatment. Thus, our study indicated that CH12 in combination with rapamycin might have a potential clinical application in GBM therapy in the future.

## MATERIALS AND METHODS

### Cell culture

The human glioblastoma cells U251MG and U87MG were obtained from the American Type Culture Collection. The function of PTEN is deficient in U87MG (*PTEN* deletion in exon 3) and U251MG (*PTEN* frameshift) cell lines [53–54]. U251MG and U87MG, with exogenous EGFRvIII or luciferase overexpression, were established according to previously reported methods [55]. All GBM cells were cultured in DMEM medium (Gibco, USA) supplemented with 10% fetal bovine serum (Serana, Australia) and maintained at 37°C in a humidified atmosphere of 5% CO_2_.

### Reagents

Rapamycin was purchased from Melonepharma (China). For the injections, stock rapamycin was diluted first in sterile 10% PEG400/8% ethanol and then in an equal volume of sterile 10% Tween-80 for a final concentration of 2 mg/mL [34]. The chimeric mAb CH12 (IgG1) was produced in dihydrofolate reductase-deficient CHO DG44 cells as previously described at 20 mg/mL [19].

### Western blotting analysis

The tumor tissues were surgically excised and frozen in liquid nitrogen and then homogenized in tumor lysis buffer (Prod# 78510, Thermo, USA); after centrifugation at 12,000 *g* for 10 min at 4°C, the lysates were collected. The protein was quantified using a BCA Kit (Prod# 23225, Thermo, USA), separated on SDS-PAGE gels at 8%–14% polyacrylamide according to protein weight and blotted onto a PVDF nitrocellulose membrane (Bio-Rad Laboratories, USA). The membrane was blocked in 5% milk in PBST for 1 h and then probed with primary antibodies overnight at 4°C. The following primary antibodies were used: the phosphor-EGFR, EGFR, phosphor-ERK, ERK and Bcl-xL antibodies, which were purchased from Santa Cruz Biotechnology, and the mTOR, phosphor-mTOR, phosphor-4EBP1, 4EBP1, p70S6K, phosphor-p70S6K, cleaved caspase-3, caspase-3, PARP, phosphor-AKT, AKT, Jak1, phosphor-Jak1, STAT5, phosphor-STAT5, STAT3 and phosphor-STAT3 antibodies, which were obtained from Cell Signaling Technology. After washing the membranes in PBST, they were incubated with the appropriate secondary antibodies for 1 h at room temperature, washed three times in PBST and then visualized with enhanced chemiluminescence reagent, following the manufacturer's instructions (Prod# 34080, Thermo, USA).

### Tumor xenograft studies

All mouse experiments were performed in accordance with approved protocols from Shanghai Medical Experimental Animal Care Commission. U87-EGFRvIII (8 × 10^5^) and U251-EGFRvIII cells (2 × 10^6^) in 100 μl of DMEM were injected subcutaneously in the right lateral flank of 6-week-old nude mice. When the tumor volumes reached an average of approximately 100 mm^3^, we treated the mice with vehicle control (three times per week), rapamycin (4 times per week), CH12 (three times per week) or the rapamycin-CH12 combination intraperitoneally. The tumor volumes were measured every three days in two dimensions with vernier calipers. The tumor volumes were calculated using the following formula: length × width^2^ × 0.5. When the experiment was complete, the mice were anesthetized and sacrificed by cervical dislocation. The tumors were surgically excised and weighed. Tumor tissues from the *in vivo* experiments were collected for western blot analysis and immunohistochemical studies.

### Intracranial tumor implantation

The mice were anesthetized with pentobarbital sodium and placed in a stereotaxic head frame (Kopf, Germany). The injection location was 1.5 mm anterior to the coronal suture, 2.5 mm to the right of the sagittal suture, and 3–3.5 mm below the skull. Approximately 5 × 10^5^ tumor cells tagged with luciferase in a 20-μL mixture of DMEM medium and Matrigel (1:1) were injected over 2 min using a 26–27 gauge syringe (approximately 3 mm deep), and the needle was left in position for 5 minutes and then withdrawn slowly. The muscle and skin were closed with 5–0 silk sutures. If mice showed clinical signs of distress or were moribund at any time during the surgery or recovery, the animals were euthanized by CO_2_ followed by cervical dislocation. Intracranial tumors were measured using an IVIS Spectrum-preclinical *in vivo* imaging system (Xenogen, USA).

### Immunohistochemical (IHC) analysis

To assess angiogenesis and cell proliferation in the tumors, formalin-fixed paraffin-embedded tumor tissues were immunostained using monoclonal antibodies anti-CD34 (Abcam, Cambridge, UK) and anti-Ki-67 (Santa Cruz Biotechnology, USA). After deparaffinization and rehydration, the tissue sections were incubated with 3% hydrogen peroxide in methanol to quench endogenous peroxidase. The sections were blocked for 30 min with 1% BSA and incubated with the primary antibodies at 4°C overnight. As negative controls, staining was performed in the absence of the primary antibodies. The sections were then washed with PBS and incubated with HRP-conjugated secondary antibodies for one hour. The products were then visualized using a diaminobenzidine staining kit (TIANGEN Biotech, Beijing, China) and counterstained in hematoxylin.

As a measure of proliferation, the Ki-67 labeling index was determined as the ratio of (labeled nuclei)/(total nuclei) in high power fields (200 ×). Approximately 2,000 nuclei were counted in each case by systematic random sampling.

The microvessel density (MVD) was determined by measuring the number of stained microvessels in each section from six mice of each group as described [57]. The mean microvessel count of the six most vascular areas was taken as the MVD, which was expressed as the absolute number of microvessels per 0.74 mm^2^(200 × field).

### TUNEL assay

The terminal deoxynucleotidyl transferase dUTP nick-end labeling (TUNEL) assay was performed according to kit instructions (Qia39, Merck, USA). The tumor tissue sections were deparaffinized, rehydrated and incubated with proteinase K (20 μg/mL) for 20 min at 37°C. After several washes with TBS, the specimen was covered in 1 × equilibration buffer for 30 min and then incubated with a mixture of 57.0 μL of Fluorescein-FragEL^™^ TdT Labeling Reaction Mix and 3 μL of TdT Enzyme for 1.5 h at 37°C in the dark. Then, the slides were rinsed in TBS three times. A glass coverslips were mounted using Fluorescein-FragEL^™^ Mounting Media, and the slides were visualized under a fluorescence microscope (OLYMPUS IX71, Japan). TUNEL-positive cells were counted at 200 × magnification. The apoptotic index was calculated as a ratio of (apoptotic cell number)/(total cell number) in each field.

### Evaluation of the combination effect

The coefficient of drug interaction (CDI) was used to evaluate the combination effect. In the *in vivo* tumor xenograft model, endpoint tumor sizes were analyzed for a combination effect using the formula CDI = (AB/C)/(A/C × B/C), where C is the tumor volume of the vehicle group, A or B is the tumor volume of the antibody monotherapy group, and AB is the tumor volume of the combination group. CDI values < 1, = 1 or > 1 indicate that the combination is synergistic, additive or antagonistic, respectively [58–59].

### Statistical analysis

ANOVA (one-way analysis of variance) and the Student's *t*-test were used to analyze significant differences between groups under the different conditions. *P* < 0.05 was considered a statistically significant difference.

## SUPPLEMENTARY MATERIALS FIGURES



## References

[1] Ostrom QT, Gittleman H, Farah P, Ondracek A, Chen Y, Wolinsky Y, Stroup NE, Kruchko C, Barnholtz-Sloan JS (2013). CBTRUS statistical report: Primary brain and central nervous system tumors diagnosed in the United States in 2006–2010. Neuro Oncol.

[2] Maher EA, Furnari FB, Bachoo RM, Rowitch DH, Louis DN, Cavenee WK, DePinho RA (2001). Malignant glioma: genetics and biology of a grave matter. Genes Dev.

[3] Stupp R, Mason WP, van den Bent MJ, Weller M, Fisher B, Taphoorn MJ, Belanger K, Brandes AA, Marosi C, Bogdahn U, Curschmann J, Janzer RC, Ludwin SK (2005). Radiotherapy plus concomitant and adjuvant temozolomide for glioblastoma. N Engl J Med.

[4] Omuro A, DeAngelis LM (2013). Glioblastoma and other malignant gliomas: a clinical review. JAMA.

[5] Gilbert MR, Wang M, Aldape KD, Stupp R, Hegi ME, Jaeckle KA, Armstrong TS, Wefel JS, Won M, Blumenthal DT, Mahajan A, Schultz CJ, Erridge S (2013). Dose-dense temozolomide for newly diagnosed glioblastoma: a randomized phase III clinical trial. J Clin Oncol.

[6] Karcher S, Steiner HH, Ahmadi R, Zoubaa S, Vasvari G, Bauer H, Unterberg A, Herold-Mende C (2006). Different angiogenic phenotypes in primary and secondary glioblastomas. Int J Cancer.

[7] Kabat GC, Etgen AM, Rohan TE (2010). Do steroid hormones play a role in the etiology of glioma?. Cancer Epidemiol Biomarkers Prev.

[8] Tso CL, Freije WA, Day A, Chen Z, Merriman B, Perlina A, Lee Y, Dia EQ, Yoshimoto K, Mischel PS, Liau LM, Cloughesy TF, Nelson SF (2006). Distinct transcription profiles of primary and secondary glioblastoma subgroups. Cancer Res.

[9] Maher EA, Brennan C, Wen PY, Durso L, Ligon KL, Richardson A, Khatry D, Feng B, Sinha R, Louis DN, Quackenbush J, Black PM, Chin L (2006). Marked genomic differences characterize primary and secondary glioblastoma subtypes and identify two distinct molecular and clinical secondary glioblastoma entities. Cancer Res.

[10] Brennan CW, Verhaak RG, McKenna A, Campos B, Noushmehr H, Salama SR, Zheng S, Chakravarty D, Sanborn JZ, Berman SH, Beroukhim R, Bernard B, Wu CJ (2013). The somatic genomic landscape of glioblastoma. Cell.

[11] Pelloski CE, Ballman KV, Furth AF, Zhang L, Lin E, Sulman EP, Bhat K, McDonald JM, Yung WK, Colman H, Woo SY, Heimberger AB, Suki D (2007). Epidermal growth factor receptor variant III status defines clinically distinct subtypes of glioblastoma. J Clin Oncol.

[12] Del VC, Giacomini CP, Vogel H, Jensen KC, Florio T, Merlo A, Pollack JR, Wong AJ (2013). EGFRvIII gene rearrangement is an early event in glioblastoma tumorigenesis and expression defines a hierarchy modulated by epigenetic mechanisms. Oncogene.

[13] Huang HS, Nagane M, Klingbeil CK, Lin H, Nishikawa R, Ji XD, Huang CM, Gill GN, Wiley HS, Cavenee WK (1997). The enhanced tumorigenic activity of a mutant epidermal growth factor receptor common in human cancers is mediated by threshold levels of constitutive tyrosine phosphorylation and unattenuated signaling. J Biol Chem.

[14] Schmidt MH, Furnari FB, Cavenee WK, Bogler O (2003). Epidermal growth factor receptor signaling intensity determines intracellular protein interactions, ubiquitination, and internalization. Proc Natl Acad Sci U S A.

[15] Garcia DPI, Adams GP, Sundareshan P, Wong AJ, Testa JR, Bigner DD, Weiner LM (1993). Expression of mutated epidermal growth factor receptor by non-small cell lung carcinomas. Cancer Res.

[16] Moscatello DK, Holgado-Madruga M, Godwin AK, Ramirez G, Gunn G, Zoltick PW, Biegel JA, Hayes RL, Wong AJ (1995). Frequent expression of a mutant epidermal growth factor receptor in multiple human tumors. Cancer Res.

[17] Shinojima N, Tada K, Shiraishi S, Kamiryo T, Kochi M, Nakamura H, Makino K, Saya H, Hirano H, Kuratsu J, Oka K, Ishimaru Y, Ushio Y (2003). Prognostic value of epidermal growth factor receptor in patients with glioblastoma multiforme. Cancer Res.

[18] Batra SK, Castelino-Prabhu S, Wikstrand CJ, Zhu X, Humphrey PA, Friedman HS, Bigner DD (1995). Epidermal growth factor ligand-independent, unregulated, cell-transforming potential of a naturally occurring human mutant EGFRvIII gene. Cell Growth Differ.

[19] Jiang H, Wang H, Tan Z, Hu S, Wang H, Shi B, Yang L, Li P, Gu J, Wang H, Li Z (2011). Growth suppression of human hepatocellular carcinoma xenografts by a monoclonal antibody CH12 directed to epidermal growth factor receptor variant III. J Biol Chem.

[20] Mishima K, Johns TG, Luwor RB, Scott AM, Stockert E, Jungbluth AA, Ji XD, Suvarna P, Voland JR, Old LJ, Huang HJ, Cavenee WK (2001). Growth suppression of intracranial xenografted glioblastomas overexpressing mutant epidermal growth factor receptors by systemic administration of monoclonal antibody (mAb) 806, a novel monoclonal antibody directed to the receptor. Cancer Res.

[21] Scott AM, Lee FT, Tebbutt N, Herbertson R, Gill SS, Liu Z, Skrinos E, Murone C, Saunder TH, Chappell B, Papenfuss AT, Poon AM, Hopkins W (2007). A phase I clinical trial with monoclonal antibody ch806 targeting transitional state and mutant epidermal growth factor receptors. Proc Natl Acad Sci U S A.

[22] Salmena L, Carracedo A, Pandolfi PP (2008). Tenets of PTEN tumor suppression. Cell.

[23] Chakravarti A, Zhai G, Suzuki Y, Sarkesh S, Black PM, Muzikansky A, Loeffler JS (2004). The prognostic significance of phosphatidylinositol 3-kinase pathway activation in human gliomas. J Clin Oncol.

[24] Vivanco I, Sawyers CL (2002). The phosphatidylinositol 3-Kinase AKT pathway in human cancer. Nat Rev Cancer.

[25] Kim B, Myung JK, Seo JH, Park CK, Paek SH, Kim DG, Jung HW, Park SH (2010). The clinicopathologic values of the molecules associated with the main pathogenesis of the glioblastoma. J Neurol Sci.

[26] Mellinghoff IK, Wang MY, Vivanco I, Haas-Kogan DA, Zhu S, Dia EQ, Lu KV, Yoshimoto K, Huang JH, Chute DJ, Riggs BL, Horvath S, Liau LM (2005). Molecular determinants of the response of glioblastomas to EGFR kinase inhibitors. N Engl J Med.

[27] Faivre S, Kroemer G, Raymond E (2006). Current development of mTOR inhibitors as anticancer agents. Nat Rev Drug Discov.

[28] Vogt PK (2001). PI 3-kinase, mTOR, protein synthesis and cancer. Trends Mol Med.

[29] Akhavan D, Cloughesy TF, Mischel PS (2010). mTOR signaling in glioblastoma: lessons learned from bench to bedside. Neuro Oncol.

[30] Chang SM, Wen P, Cloughesy T, Greenberg H, Schiff D, Conrad C, Fink K, Robins HI, De Angelis L, Raizer J, Hess K, Aldape K, Lamborn KR (2005). Phase II study of CCI-779 in patients with recurrent glioblastoma multiforme. Invest New Drugs.

[31] Moriggl R, Sexl V, Kenner L, Duntsch C, Stangl K, Gingras S, Hoffmeyer A, Bauer A, Piekorz R, Wang D, Bunting KD, Wagner EF, Sonneck K (2005). Stat5 tetramer formation is associated with leukemogenesis. Cancer Cell.

[32] Feng C, Cao S (2014). Activation of STAT5 contributes to proliferation in U87 human glioblastoma multiforme cells. Mol Med Rep.

[33] Latha K, Li M, Chumbalkar V, Gururaj A, Hwang Y, Dakeng S, Sawaya R, Aldape K, Cavenee WK, Bogler O, Furnari FB (2013). Nuclear EGFRvIII-STAT5b complex contributes to glioblastoma cell survival by direct activation of the Bcl-XL promoter. Int J Cancer.

[34] Neyns B, Sadones J, Joosens E, Bouttens F, Verbeke L, Baurain JF, D'Hondt L, Strauven T, Chaskis C, In'T Veld P, Michotte A, De Greve J (2009). Stratified phase II trial of cetuximab in patients with recurrent high-grade glioma. Annals of Oncology.

[35] Franceschi E, Pession A, Tallini G, Crino L, Brandes AA, Cavallo G, Magrini E, Tosoni A, Grosso D, Scopece L, Blatt V, Urbini B (2007). Gefitinib in patients with progressive high-grade gliomas: a multicentre phase II study by Gruppo Italiano Cooperativo di Neuro-Oncologia (GICNO). British Journal of Cancer.

[36] Chinot OL, Wick W, Mason W, Henriksson R, Saran F, Nishikawa R, Carpentier AF, Hoang-Xuan K, Kavan P, Cernea D, Brandes AA, Hilton M, Abrey L (2014). Bevacizumab plus radiotherapy-temozolomide for newly diagnosed glioblastoma. N Engl J Med.

[37] Mellinghoff IK, Wang MY, Vivanco I, Haas-Kogan DA, Zhu S, Dia EQ, Lu KV, Yoshimoto K, Huang JH, Chute DJ, Riggs BL, Horvath S, Liau LM (2005). Molecular determinants of the response of glioblastomas to EGFR kinase inhibitors. N Engl J Med.

[38] Sun SY, Rosenberg LM, Wang X, Zhou Z, Yue P, Fu H, Khuri FR (2005). Activation of Akt and eIF4E survival pathways by rapamycin-mediated mammalian target of rapamycin inhibition. Cancer Res.

[39] O'Reilly KE, Rojo F, She QB, Solit D, Mills GB, Smith D, Lane H, Hofmann F, Hicklin DJ, Ludwig DL, Baselga J, Rosen N (2006). mTOR inhibition induces upstream receptor tyrosine kinase signaling and activates Akt. Cancer Res.

[40] Fan QW, Cheng C, Hackett C, Feldman M, Houseman BT, Nicolaides T, Haas-Kogan D, James CD, Oakes SA, Debnath J, Shokat KM, Weiss WA (2010). Akt and autophagy cooperate to promote survival of drug-resistant glioma. Sci Signal.

[41] Britschgi A, Andraos R, Brinkhaus H, Klebba I, Romanet V, Muller U, Murakami M, Radimerski T, Bentires-Alj M (2012). JAK2/STAT5 inhibition circumvents resistance to PI3K/mTOR blockade: a rationale for cotargeting these pathways in metastatic breast cancer. Cancer Cell.

[42] Coffer PJ, Koenderman L, de Groot RP (2000). The role of STATs in myeloid differentiation and leukemia. Oncogene.

[43] Nevalainen MT, Xie J, Torhorst J, Bubendorf L, Haas P, Kononen J, Sauter G, Rui H (2004). Signal transducer and activator of transcription-5 activation and breast cancer prognosis. J Clin Oncol.

[44] Li H, Ahonen TJ, Alanen K, Xie J, LeBaron MJ, Pretlow TG, Ealley EL, Zhang Y, Nurmi M, Singh B, Martikainen PM, Nevalainen MT (2004). Activation of signal transducer and activator of transcription 5 in human prostate cancer is associated with high histological grade. Cancer Res.

[45] Liu X, Robinson GW, Gouilleux F, Groner B, Hennighausen L (1995). Cloning and expression of Stat5 and an additional homologue (Stat5b) involved in prolactin signal transduction in mouse mammary tissue. Proc Natl Acad Sci U S A.

[46] Chumbalkar V, Latha K, Hwang Y, Maywald R, Hawley L, Sawaya R, Diao L, Baggerly K, Cavenee WK, Furnari FB, Bogler O (2011). Analysis of phosphotyrosine signaling in glioblastoma identifies STAT5 as a novel downstream target of DeltaEGFR. J Proteome Res.

[47] Lee TK, Man K, Poon RT, Lo CM, Yuen AP, Ng IO, Ng KT, Leonard W, Fan ST (2006). Signal transducers and activators of transcription 5b activation enhances hepatocellular carcinoma aggressiveness through induction of epithelial-mesenchymal transition. Cancer Res.

[48] Liang QC, Xiong H, Zhao ZW, Jia D, Li WX, Qin HZ, Deng JP, Gao L, Zhang H, Gao GD (2009). Inhibition of transcription factor STAT5b suppresses proliferation, induces G1 cell cycle arrest and reduces tumor cell invasion in human glioblastoma multiforme cells. Cancer Lett.

[49] Nagane M, Levitzki A, Gazit A, Cavenee WK, Huang HJ (1998). Drug resistance of human glioblastoma cells conferred by a tumor-specific mutant epidermal growth factor receptor through modulation of Bcl-XL and caspase-3-like proteases. Proc Natl Acad Sci U S A.

[50] Li D, Ji H, Zaghlul S, McNamara K, Liang MC, Shimamura T, Kubo S, Takahashi M, Chirieac LR, Padera RF, Scott AM, Jungbluth AA, Cavenee WK (2007). Therapeutic anti-EGFR antibody 806 generates responses in murine de novo EGFR mutant-dependent lung carcinomas. J Clin Invest.

[51] Hainsworth JD, Shih KC, Shepard GC, Tillinghast GW, Brinker BT, Spigel DR (2012). Phase II study of concurrent radiation therapy, temozolomide, and bevacizumab followed by bevacizumab/everolimus as first-line treatment for patients with glioblastoma. Clin Adv Hematol Oncol.

[52] Wen PY, Chang SM, Lamborn KR, Kuhn JG, Norden AD, Cloughesy TF, Robins HI, Lieberman FS, Gilbert MR, Mehta MP, Drappatz J, Groves MD, Santagata S (2014). Phase I/II study of erlotinib and temsirolimus for patients with recurrent malignant gliomas: North American Brain Tumor Consortium trial 04–02. Neuro Oncol.

[53] Koul D, Shen R, Kim YW, Kondo Y, Lu Y, Bankson J, Ronen SM, Kirkpatrick DL, Powis G, Yung WK (2010). Cellular and *in vivo* activity of a novel PI3K inhibitor, PX-866, against human glioblastoma. Neuro Oncol.

[54] Jiang Z, Pore N, Cerniglia GJ, Mick R, Georgescu MM, Bernhard EJ, Hahn SM, Gupta AK, Maity A (2007). Phosphatase and tensin homologue deficiency in glioblastoma confers resistance to radiation and temozolomide that is reversed by the protease inhibitor nelfinavir. Cancer Res.

[55] Wang H, Jiang H, Zhou M, Xu Z, Liu S, Shi B, Yao X, Yao M, Gu J, Li Z (2009). Epidermal growth factor receptor vIII enhances tumorigenicity and resistance to 5-fluorouracil in human hepatocellular carcinoma. Cancer Lett.

[56] Eshleman JS, Carlson BL, Mladek AC, Kastner BD, Shide KL, Sarkaria JN (2002). Inhibition of the mammalian target of rapamycin sensitizes U87 xenografts to fractionated radiation therapy. Cancer Res.

[57] Poon RT, Ng IO, Lau C, Yu WC, Yang ZF, Fan ST, Wong J (2002). Tumor microvessel density as a predictor of recurrence after resection of hepatocellular carcinoma: a prospective study. J Clin Oncol.

[58] Cao SS, Zhen YS (1989). Potentiation of antimetabolite antitumor activity *in vivo* by dipyridamole and amphotericin B. Cancer Chemother Pharmacol.

[59] Clarke R (1997). Issues in experimental design and endpoint analysis in the study of experimental cytotoxic agents *in vivo* in breast cancer and other models. Breast Cancer Res Treat.

