# Supplementary material for: Combination of an anti-EGFRvIII antibody CH12 with Rapamycin synergistically inhibits the growth of EGFRvIII+PTEN− glioblastoma in vivo
Source: Oncotarget. 2016 Mar 26;7(17):24752–65. doi: 10.18632/oncotarget.8407 (PMC5029739; doi:10.18632/oncotarget.8407)
Supplement: Supplementary file 1 [file oncotarget-07-24752-s001.pdf]

Combination of an anti-EGFRvIII antibody CH12 with Rapamycin synergistically inhibits the growth of EGFRvIII^{+}PTEN^{-} glioblastoma *in vivo*

Supplementary Materials

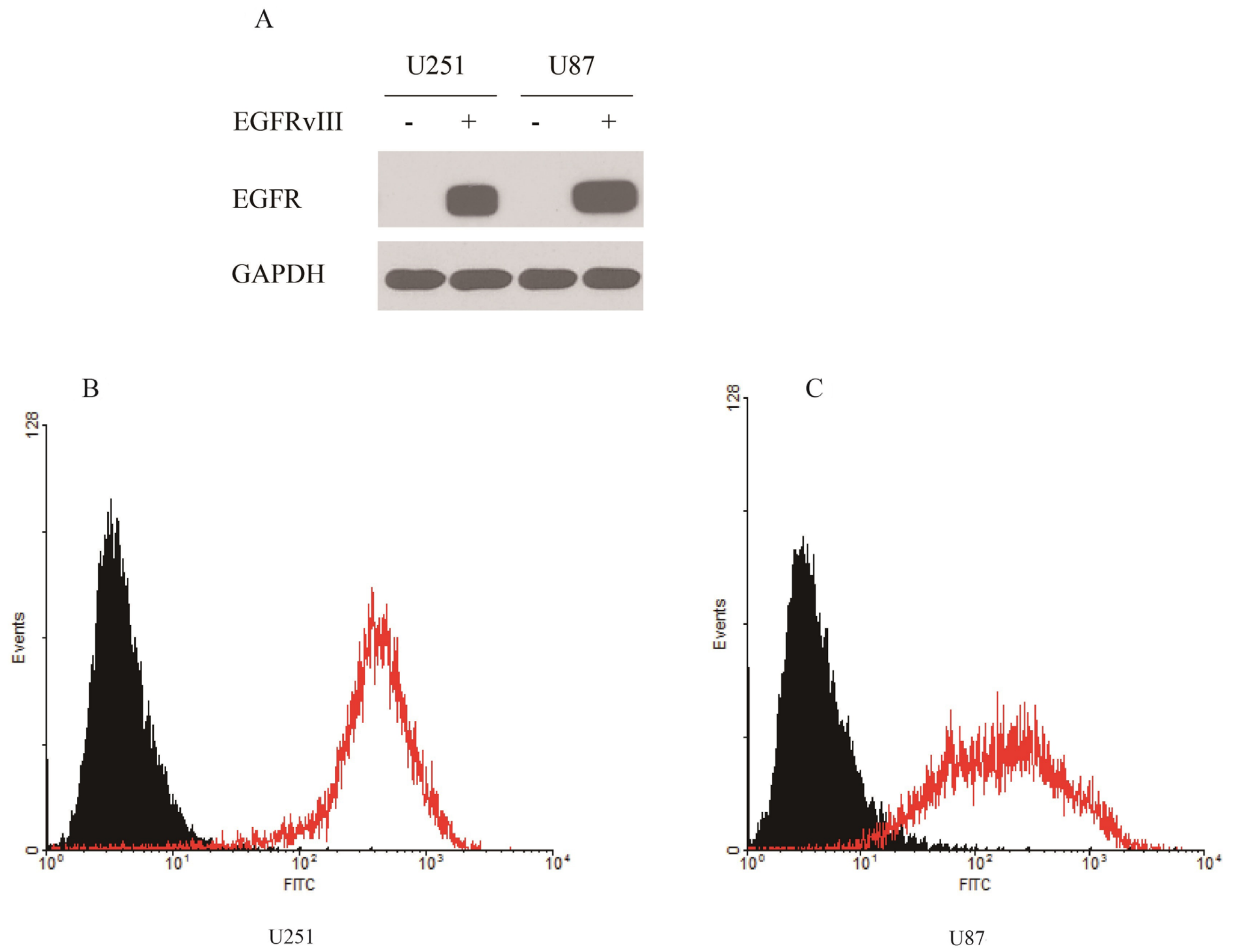

Supplementary Figure S1: U251 and U87 cells with EGFRvIII overexpression was constructed and had the binding capacity of CH12. **(A)** Western blot assay to analysis EGFRvIII expression in U251 and U87 cell lines after infected with virus carried with EGFRvIII plasmid and screened the EGFRvIII high expression clone. **(B)** The binding capacity of CH12 to EGFRvIII^{+} U251 cell lines was determined by FACS assay. Blank: parent cells, red: EGFRvIII overexpressing cells. **(C)** The binding capacity of CH12 to EGFRvIII^{+} U87 cell lines was determined by FACS assay. Blank: parent cells, red: EGFRvIII overexpressing cells.

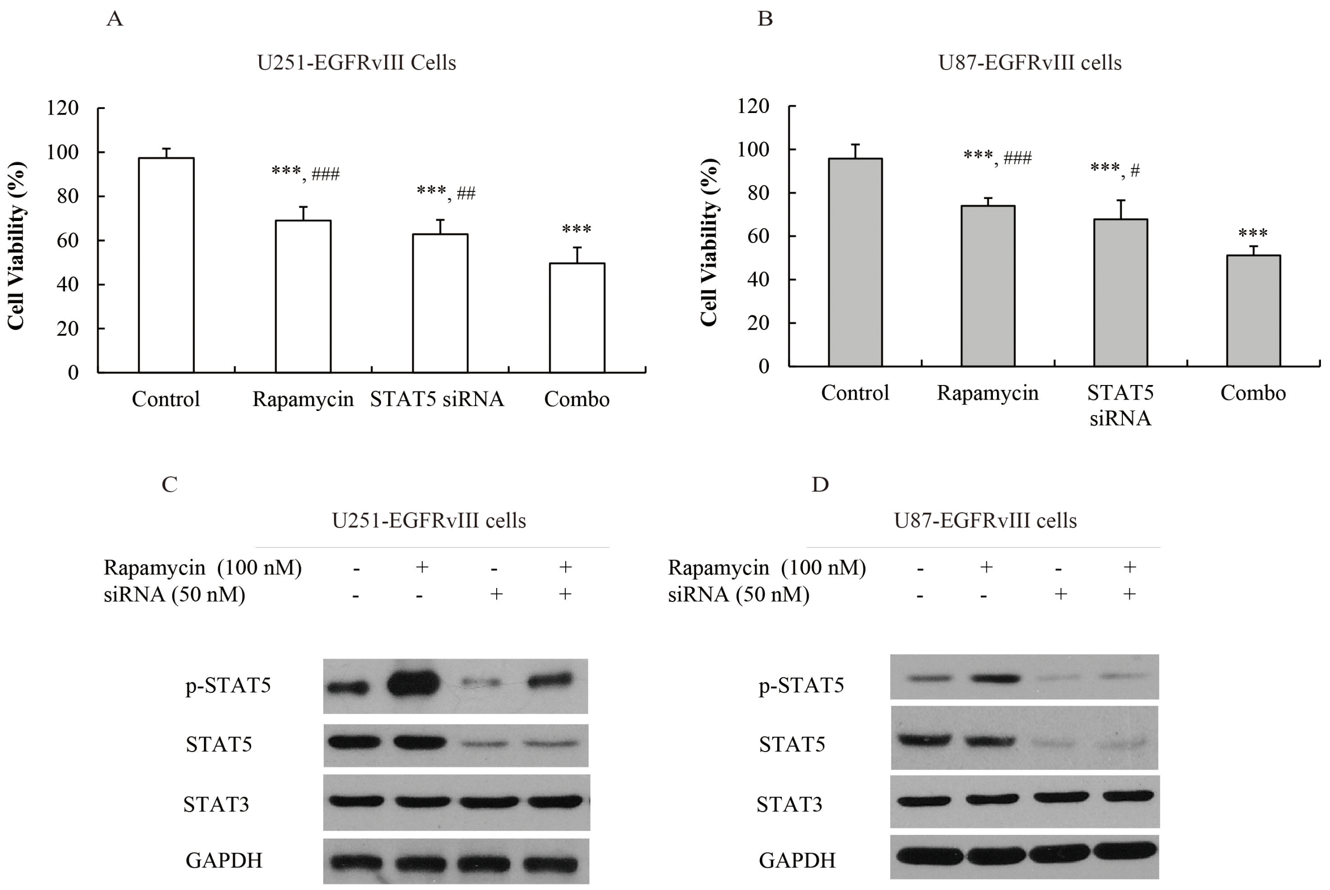

Supplementary Figure S2: Rapamycin in combination with STAT5 siRNA increased the antitumor efficacy of Rapamycin in U251-EGFRvIII and U87-EGFRvIII cells. (A, B) CCK-8 assay examining the effect of STAT5 siRNA in combination with Rapamycin in U251-EGFRvIII and U87-EGFRvIII cells. U251-EGFRvIII (A) and U87-EGFRvIII (B) cells were exposed to Rapamycin at a concentration of 100 nM or STAT5 siRNA at a concentration of 50 nM or the combination for 48 hours. Data are expressed as the cell viability in triplicate experiments (Bars, SD). Statistical significance is indicated $^{*}P < 0.05$, $^{**}P < 0.01$, $^{***}P < 0.001$ versus control group, $^{\#}P < 0.05$, $^{\#\#}P < 0.01$, $^{\#\#\#}P < 0.001$ versus combo group. **(C, D)** Immunoblots assessing signaling events upon treatment with STAT5 siRNA, Rapamycin or the combination in U251-EGFRvIII and U87-EGFRvIII cells.
